# Challenges and opportunities in cell expansion for cultivated meat

**DOI:** 10.3389/fnut.2024.1315555

**Published:** 2024-02-07

**Authors:** Michelle Hauser, Amit Zirman, Roni Rak, Iftach Nachman

**Affiliations:** ^1^The George S. Wise Faculty of Life Sciences, Tel Aviv University, Tel Aviv, Israel; ^2^Institute for Animal Research, Agricultural Research Organization, Volcani Center, Rishon LeZion, Israel

**Keywords:** *in vitro* cultivation, proliferation, self-renewal, cell cylce, genetic regulation

## Abstract

The cultivation of meat using *in vitro* grown animal stem cells offers a promising solution to pressing global concerns around climate change, ethical considerations, and public health. However, cultivated meat introduces an unprecedented necessity: the generation of mass scales of cellular biomaterial, achieved by fostering cell proliferation within bioreactors. Existing methods for *in vitro* cell proliferation encounter substantial challenges in terms of both scalability and economic viability. Within this perspective, we discuss the current landscape of cell proliferation optimization, focusing on approaches pertinent to cellular agriculture. We examine the mechanisms governing proliferation rates, while also addressing intrinsic and conditional rate limitations. Furthermore, we expound upon prospective strategies that could lead to a significant enhancement of the overall scalability and cost-efficiency of the cell proliferation phase within the cultivated meat production process. By exploring knowledge from basic cell cycle studies, pathological contexts and tissue engineering, we may identify innovative solutions toward optimizing cell expansion.

## Introduction

Cultivated meat (CM) represents a revolutionary paradigm in the realm of sustainable and ethical food production. Unlike traditional methods of meat production, which involve raising and slaughtering animals, cultivated meat is produced by growing animal cells *in vitro*. This process involves cultivating animal cells in a controlled *in vitro* environment and differentiating them into specific cell types, such as muscle and adipose cells. These cells are then arranged into a final product that aims to be nutritionally and sensorially similar to conventionally produced meat (1, 2), which is not the case yet (3, 4).

For CM technology to effectively integrate into the market and substitute a significant portion of conventional meat, it is imperative that the bioprocess demonstrates the capacity to efficiently generate meat products on a large scale while remaining economically competitive within the food industry (5). This unprecedented demand for both scalability and affordability calls for innovative bioprocessing solutions, as until recently, research on *in vitro* cell culture has primarily centered around research lab scales and biomedical applications, which typically involve significantly smaller biomass requirements.

A significant challenge that currently impedes the mass production of cultured meat is the limited proliferative capacity and rate of the initial cell population. This phase marks the outset of the production process, where a small cell sample is expanded into a robust and viable cell population. The proliferation stage is of paramount importance as it wields a direct impact on the efficiency and scalability of the entire downstream production process: it serves as the foundation for the entire manufacturing process, upon which the differentiation and processing of the final product depend (6).

**Figure 1:**
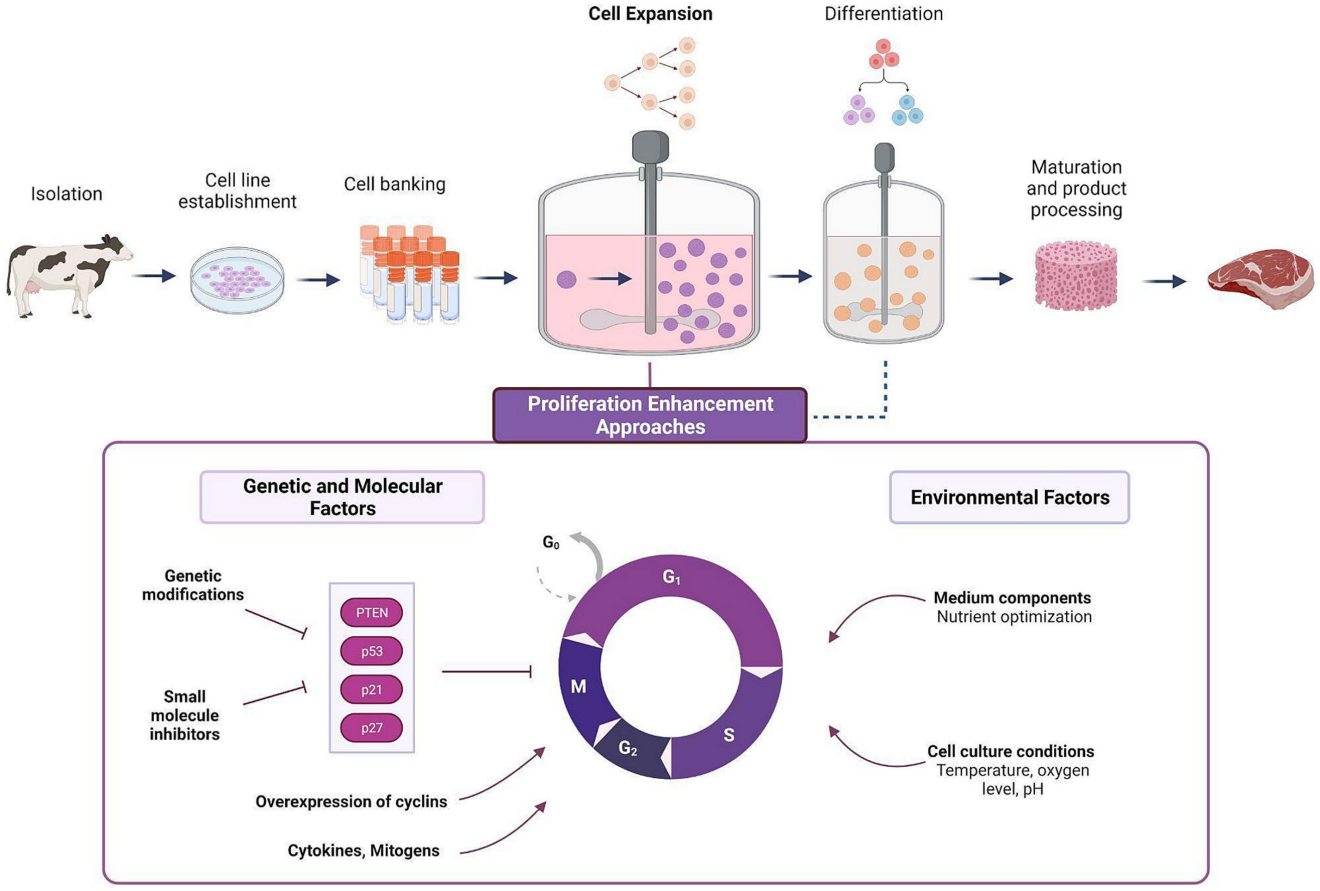
Cell expansion stage in a typical CM production process and the various approaches for proliferation enhancement. In the cell expansion stage, a small sample of cells are made to proliferate into large biomasses, followed by cell differentiation and tissue maturation (i.e., muscle fiber formation, or lipid accumulation). Various approaches can be adopted to enhance the efficiency of the expansion stage, ranging from the optimization of environmental factors, and through a more direct manipulation of genetic and molecular factors involved in the regulation of cell division (see Table 1).

Subsequent to the cell proliferation phase, the expanded cells undergo a process wherein they transform into one or more specific cell types, such as muscle, fat, and connective tissue cells, similar to those found in traditional meat. The particular treatment required for cell differentiation depends significantly on the original cell type and the intended cell fate. Subsequently, the differentiated cells are processed and matured toward the desired tissue composition (muscle fiber formation, fat accumulation). Following this, the grown tissue undergoes further processing to ultimately become the final meat product, similar to the process of conventional muscle to meat conversion [reviewed in (7)]. This post-mortem process involves various biological mechanisms such as pH drop, activation of enzymes related to proteolysis, and Maillard reaction (Figure 1).

We explore the complexities inherent in the cell proliferation phase within CM production and discuss the various aspects that encompass this pivotal stage. Our objective is to illuminate the noteworthy strides, obstacles, and prospects that characterize this critical juncture in the evolution of CM.

### Proliferation efficiency, scalability and costs

In order to produce CM, the mechanism by which a small number of cells (typically 10^5^–10^6^) are induced to multiply into a vast biomass (typically >10^13^ cells) must be extremely efficient and economical. Enhancing proliferation efficiency can cut growth factor and medium expenses, enhancing cost-effectiveness and scalability. While enhanced proliferation rate might change consumption of media ingredients such as glucose and contribute to waste production (8, 9), other media components may be less affected (10), leading to overall reduction in resource usage. Hence, optimized growth rates can use less energy, nutrients, and materials, allow shorter production cycles, thus promoting sustainability and the potential to mitigate the environmental burden associated with conventional livestock farming.

**Figure 2:**
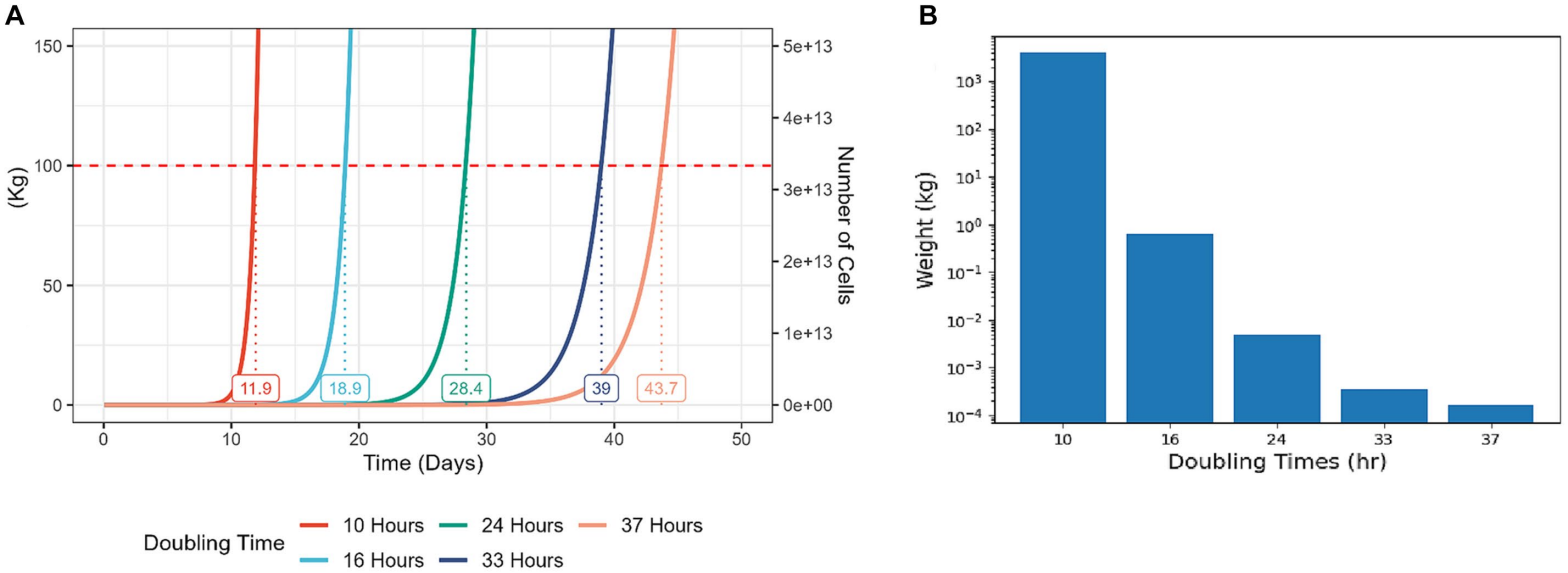
Modest increases in cell proliferation rates result in a significantly higher yield at a given time period. Cell growth simulations starting with 100,000 cells and assuming weight of 3 ng per cell, differing in proliferation rate. In each case, constant proliferation rate is assumed, and no Hayflick limit, ignoring cell death or differentiation events. **(A)** A representation of total cell yield weight vs. culture time in days. Each line on the graph represents the growth of the cell population with the corresponding doubling time. The numbers on the graph indicate the predicted time for the total cell weight to reach 100 kg. **(B)** A representation of total cell yield weight of populations with different doubling times, during a culture period of 14 days. For reference, typical doubling times of bESCs and bMSCs are 24–70 h (14), chicken fibroblasts can be optimized to divide every 20 h (15) and the fastest doubling times for mammalian cells are 5–10 h [T cells, (16, 17)].

Because cell proliferation is an inherently exponential process, even modest improvements to cell proliferation rates can yield significant cost reductions for the process of CM production (Figure 2). Strategies for enhancing cell proliferation can be divided to manipulation of external conditions or cell-internal genetic and molecular factors (Figure 1, bottom). To date, much effort in this challenge focused on medium optimization and external conditions such as temperature and oxygen levels (11–13). Developing new methods to enhance cell proliferation can be highly beneficial, and this article explores potential strategies for achieving that goal.

### Constraints and mechanisms of cell proliferation rates

The regulation over rate of proliferation is intricately controlled by a highly conserved network of cell cycle regulators. These regulators coordinate the progression through different phases of the cell cycle through the involvement of distinct signaling pathways. Cell cycle regulators play critical roles in various physiological and pathological processes, such as cancer, tissue regeneration, and development (14). Two families of negative regulators called cyclin-dependent kinase (CDK) inhibitors mediate cell cycle arrest though their expression: CIP/KIP (p21, p57, and p27) and INK4 (p15, p16, p18, and p14). Other signaling pathways are also involved in cell cycle progression regulation. For example, PTEN (Phosphatase and tensin homolog), is a known tumor suppressor gene that normally blocks the G1-S and G2-M transitions to avoid premature mitosis and DNA replication beginning. PTEN also governs the start and stop of the cell cycle by regulating the induction of senescence and upholding cellular quiescence (15) (Table 1). Recent studies have provided evidence that cell cycle regulators can modulate the rate of stem cell self-renewal both *in vivo* and *in vitro*. For example, research has shown that the CDK inhibitor p21 plays a crucial role in controlling the proliferation of forebrain neural stem cells by reducing cell cycle arrest in *in vitro* settings, and its loss compromises their relative quiescence (16).

**Table 1:** Cell cycle regulators role in proliferation.

Common name	Gene name	Role in cell growth	Effect of inhibition on proliferation
P21/CIP1	Cyclin-dependent kinase inhibitor 1A (*CDKN1A*)	The encoded protein binds to and inhibits the activity of cyclin-dependent kinase2 or cyclin-dependent kinase4 complexes, and thus functions as a regulator of cell cycle progression at G1. Its expression is regulated by p53 (21)	Cdkn1a deletion elevates proliferation and osteogenic differentiation of bone marrow mesenchymal stem cells (21)
p27/Kip1	Cyclin-dependent kinase inhibitor 1B (*CDKN1B*)	Controls the progression from G1 to the S phase of the cell cycle in response to both mitogenic and anti-mitogenic stimuli (19).	No information
PTEN	Phosphatase and tensin homolog (*PTEN*)	Controls cell proliferation and survival by regulating cell cycle phases, including the G1/S and G2/M transitions. Involved in genetic transmission during cell cycle, promotes the fidelity of DNA replication and chromosome segregation (19)	PTEN-KO in BMSCs showed an increased proliferation capability but decreased multi-directional differentiation potential (22).
P53	Tumor protein p53 (*TP53*)	p53 is a transcription factor that protects cells against stress, by modulating genes that induce growth arrest, repair, apoptosis, senescence or altered metabolism (23).	MSCs derived from p53KO mice showed an augmented proliferation rate and a shorter doubling time (24). Spontaneous immortalization of chicken fibroblasts resulted in downregulation of TP53 (15)
p15/p16/INK4	Cyclin-dependent kinase inhibitor 2A/B (*CDKN2A/B*)	Those genes encode a cyclin-dependent kinase inhibitor, which forms a complex with CDK4/6, and prevents the activation of the CDK kinases. Thus, the encoded protein functions as a cell growth regulator that controls cell cycle G1 progression.	Loss of CDKN2B accelerates SMC proliferation while paradoxically increases vascular apoptosis, due to interaction with the MDM2-p53 pathway (23). CRISPR/Cas9 disruption of p15 in chicken muscle SC increases proliferation capacity (29)

The research of these systems has yielded a wealth of information over the past few decades, with the aim of minimizing the pathological effects of loss of regulatory function that lead to faster and uncontrolled proliferation. While some literature is available on using this knowledge for the opposite goal of enhancing cell proliferation for tissue engineering purposes (17, 18), CM presents an unprecedented challenge in cell expansion requirements, that calls for leveraging this abundance of knowledge for the enhancement of cell proliferation rate in a controlled manner.

*In vivo*, the cell cycle duration for a specific cell type depends on multiple factors. Molecularly, each phase must allow sufficient time for DNA replication and error correction while maintaining genomic stability (19). At the cellular level, cells must reach a certain size and generate specific molecular machinery, mainly in G1 (20). At the tissue level, cell division rates must match tissue growth rates and synchronize with neighboring tissues (21). However, these constraints, especially the latter, may not apply during *in vitro* cell mass growth for purposes like CM. Unlike in an organism, where tight growth synchronization and size control are crucial for viability, in CM these considerations become irrelevant. This presents an opportunity to expedite the cell cycle while preserving desired cell properties. Manipulating cell cycle regulators and tumor suppressor genes can accelerate *in vitro* stem cell proliferation without compromising genomic integrity and differentiation potential. While genetic manipulations of such regulators have been suggested in the context of extending self-renewal capacity, or for invoking proliferation in otherwise senescent cells, less attention has been devoted to shortening the cell cycle duration (22, 23).

Unlike bacterial cell proliferation, animal stem cells have not evolved for maximum proliferation rates. This raises fundamental, rather unexplored questions: What is the highest viable proliferation rate for animal cells that maintains potency and genomic integrity? Do cells in standard *in vitro* conditions maintain a “safety margin” in their division rates or do they eventually reach maximal rates? How does this limit vary among cell types? To what extent does the culturing method (i.e., cells adhered to micro-beads or other surfaces vs. cells grown in suspension) dictate cell proliferation rate? Answers to these questions could significantly affect the scalability of CM.

In the context of stem cells, there’s a tradeoff between proliferation and differentiation. In mammals, most adult tissue cells are specialized and non-dividing, except for stem cells (24, 25). Proliferating and differentiated cells differ in gene expression and translation mechanisms (26, 27). Furthermore, the transition between the states is tightly regulated and natural systems reveal optimized strategies for the timely coordination of proliferation and differentiation (28). Animal cell biology reflects evolutionary pressures at cellular and organismal levels, which can sometimes conflict, like fast growth being associated with cancer (29). Engineering and evolving cells in culture may bypass these trade-offs and reveal new pathways for proliferation. Unlike cancer, where gene overexpression or silencing is constant, genetic engineering or small molecule strategies can transiently modulate gene expression, allowing cells to retain differentiation potential.

### Strategies for mitigating cellular replicative challenges - learning from cancer and immunity

Considerable knowledge has been amassed regarding enhanced cell proliferation in pathological contexts like cancer. Mass cell expansion in CM exhibits distinctive features that, while differing from classical cancer hallmarks, can draw inspiration from certain drivers and mechanisms found in cancer cell proliferation (30). Both CM cells and cancer cells must sustain proliferation signaling, evade growth suppression, resist cell death, and possibly achieve replicative immortality. Examples for sustained proliferation learned from cancer progression include the support of mitogenesis by growth factors, over-expression of the growth factor and the receptor, as well as mutations at the cytoplasmic tyrosine kinase domain, all of which contribute to constitutive proliferation (31, 32). In addition, cancer cells were also found to reduce dependency on growth factors regulation by activation of signals downstream to the receptors, as well as inhibition of associated negative feed-back loops. Example for such inhibition is PTEN loss of function mutation, which amplifies tyrosine kinase signaling (PI3K). The knowledge accumulated from cancer research can be utilized to support sustained proliferation by growth factor addition to the media, or by direct targeting of proliferation related genes identified in cancer progression,

There are, however, important distinctions between CM cells and cancer cells. Unlike in cancer, CM cells do not need to evade the immune response, and inducing vascularity is unnecessary in most approaches, although might be relevant in “whole cut” product approach. The most significant difference lies in genome stability and differentiation potential. While cancer is marked by genomic instability and loss of differentiation markers, cultured meat cells must maintain genomic stability for consistent meat production (33, 34). Additionally, preserving differentiation potential is crucial for cultured meat cells to develop into the desired muscle or fat tissues mimicking conventional meat.

Another process reminiscent of the need for extensive proliferation is the T-cell expansion phase triggered by antigen stimulation. For example, naïve T cells become activated when encountering antigen-presenting cells, leading to rapid proliferation known as the expansion phase. In this phase, these cells exhibit one of the highest doubling rates among mammalian cells, ranging from 5 to 8 h, and in some cases, a peak doubling time as short as 4.5 h has been reported (35). While T cells are significantly different from multipotent stem cells in size, survival and function (36), investigating the genetic factors behind this remarkable proliferation rate may reveal new targets for controlling proliferation and expediting the cell cycle (37).

### Approaches for enhanced cell expansion

Efforts to improve cell expansion efficiency can be categorized into two primary approaches. First, by increasing cell yield via cell viability or reducing cell death. Second, by boosting the overall proliferation activity of cells, which involves generating more cells from a single cell by shortening the cell cycle duration or manipulating the fraction of cells entering the cell cycle.

Various strategies to enhance cell expansion efficiency can be employed, including well-established practices in cell culture and innovative methods. These interventions can be grouped into two categories: those related to environmental factors, such as the optimization of nutrients, and those involving genetic or molecular manipulations directly targeting cell division processes. Genetic modifications can focus on genes that limit replication, while the medium can be enriched with biochemical factors like growth factors, cytokines, mitogens, or other small molecules to stimulate proliferation. We next focus on novel strategies that tackle cell proliferation limitations in a direct manner.

#### Chemical interventions for increased proliferation

Harnessing chemical stimulation to enhance cell proliferation represents a potentially powerful avenue in CM production. By employing small molecules that can selectively modulate key cell cycle regulators or rate limiting proteins, precise control over cellular growth and division could be exerted (38, 39). For example, the presence of a small molecule inhibitor of p21 in cell growth medium, results in an enhancement in cell proliferations (17). In a similar manner, small molecules targeted at other negative cell cycle regulators such as p27, PTEN and others can be employed. This approach could offer a straightforward means of influencing cell proliferation rates and achieving higher than usual production yields, in a controlled manner - inhibitors could be added while cells undergo the expansion phase and removed from the medium in later steps of the process such as differentiation. However, the utilization of chemical interventions in this context is not without challenges. While they might expedite the process, concerns about food safety and the broader impact of these chemicals on cell behavior and product quality are valid considerations that require rigorous assessment and thorough testing.

#### Genetic modification approaches for increased proliferation

Enhancing cell proliferation through genetic engineering is another promising avenue to explore. Manipulating specific genes can reduce proliferation time, immortalize cells, and enable adaptation to diverse conditions, such as 3D culturing and various media compositions. Precisely targeting a single gene can significantly reduce off-target effects while achieving comparable or improved outcomes. This approach was recently explored by Upside Foods, Inc., demonstrating an increased growth rate of cells by CRISPR knockout or inhibition of CDK inhibitor genes (p. 16 and p. 15) (22). Selecting which gene to edit demands careful consideration, focusing on maximizing positive proliferation effects and minimizing undesirable consequences. One approach involves altering genes perturbed in cancer (e.g., TP53, PTEN). Such alteration can be achieved spontaneously by prolonged culturing (33), or by directed targeting. Alternatively, genes related to proliferation pathways like MAPK, NF-κB signaling, and WNT pathways can be chosen. An increasingly popular method involves high throughput screening using CRISPR, enabling the assessment of many candidates, ranging from dozens to genome-wide scales (40).

However, this approach warrants mindful deliberation. The stable expansion of genetically engineered cells over time, while maintaining required abilities and characteristics such as differentiation potential is non-obvious and requires validation. Moreover, while generally deemed safe for consumption by experts, and though recently approved by the US Food and Drug Administration in the context of TERT-immortalized chicken cells (Cell Culture Consultation (CCC) 000002), genetically engineered organisms (GMOs) still spark debates about public acceptance (41). Indeed, many factors depend on the chosen genetic engineering technique (Boxes 1, 2).

**BOX 1 Gene editing techniques for improved cell lines.**

To safely and effectively transform cells, two main considerations should be addressed: editing strategy, and delivery method. Editing involves selecting the editing tool and determining whether the expression will be transient or permanent. In recent years we saw the rise of the popular CRISPR-Cas9 system, allowing for versatile, efficient editing across various organisms. Several CRISPR sub-systems exists, distinguished by their target molecule (Cas9 for DNA or CasRX for RNA) and by their mode of action (knockdown by editing, CRISPRi for transient gene inhibition, or CRIPSRa for transient gene activation). This system, highly efficient and adaptable, remains the premier choice for genetic manipulation. Alternative methods exist for transient manipulation like mRNA delivery and RNAi. mRNA delivery, popularized by Covid-19 vaccine, delivers mRNA molecules, which transiently encode into a protein that can enhance processes such as cell proliferation. RNAi (RNA interference) similarly modulates gene expression via distinct targeting and mRNA degradation mechanisms.

Next, we have to choose a delivery method by their ease of use, efficiency and safety. Two classic approaches to vector delivery involve chemical transduction and electroporation. Both aim to aid plasmid vector passage through cell membranes–via lipid encapsulation (chemical) or membrane pore creation through electricity (electroporation). While efficient for transient gene expression, they prove inefficient for genetic engineering, given stochastic genetic integration. The second group of tools used is virus transduction, particularly lentivirus and adenovirus. Lentiviral particles carry a plasmid vector that integrates into the host genome. In addition, lentiviruses can be pseudotyped with different envelope proteins that affect their tropism and change the range of cells they can transduce. This method is very efficient and scalable, but requires BSL-2 level biohazard precautions. Adenovirus offers a comparable approach, exerting transient effects without genomic integration, and is considered safer for use as it is not known to cause any diseases in humans. Adenovirus excels in enriching titer through multiple rounds of transduction and accommodating larger vectors (up to 30 kb compared to lentiviruses ~10kbp). While CRISPR-Cas9 editing via lentivirus delivery seems the most promising avenue, both editing and delivery techniques should be carefully chosen based on the needs for cultured meat.

**BOX 2 Replicative limits and immortalization**

To achieve cost-effective cultured meat, it’s essential to enhance both cell replication speed and the overall number of replications (42). While certain cell sources, like PSCs, exhibit indefinite division under suitable growth conditions, others have limited doubling and eventually enter senescence. Immortalization offers an avenue to bypass this state. Initial success came with immortalized HeLa cells, followed by numerous other cell lines that were induced for immortalization via varied genetic manipulation. Immortalization can be attained by dysregulating the cell cycle either through viral transgene expression (e.g., SV40 antigen) or by suppressing cell cycle-related genes (e.g., TP53, MYC, Rb, and Ras).However, the most commonly used method for immortalization is by expressing telomerase reverse transcriptase protein (TERT). Hence, the possibility arises to indefinitely sustain MSCs, satellite cells or fibroblasts through immortalization, a feat achieved by several independent research groups.

However, immortalization brings forth challenges; Prolonged culturing invariably introduces genetic mutations that modify cellular biology and function. Thus, generating immortalized cell lines must be coupled with monitoring their functionality (e.g., differentiation capacity) over extended culturing periods. Nonetheless, this strategy offers a straightforward approach to substantially amplify the cell yield from each isolated batch.

## Conclusion

The successful realization of CM hinges on the efficient proliferation and expansion of animal cells in controlled environments. This novel need for production scales raises intriguing biological questions that demand exploration. One of the fundamental questions is centered around the lower limit of cell division time: How fast can we coax animal cells to multiply, and does this timeframe vary among different cell types? Understanding these limits can guide efforts to push cells to divide as close to this limit as possible, thereby optimizing the production process and minimizing resource consumption.

However, in our quest for increased cell proliferation, we must not lose sight of the critical safety, regulatory, and quality control aspects. Ensuring that CM is not only sustainable and scalable but also safe and reliable is of paramount importance. Of note, though the approaches we describe here are mostly independent of the cell growth conditions (suspension vs. adherence, serum-based vs. serum-free, bioreactor shear forces, etc.), there may be interactions with these parameters. Moreover, faster proliferation may have a compound effect on DNA damage accrued over the multiple population doublings, depending on the speedup mechanism employed. For example, mechanisms that speed up events around DNA replication or related checkpoints may raise the likelihood of un-corrected damage events. On the other hand, shortening of G1 phase may reduce the exposure time to environmental damage. Of course, the extent and nature of DNA damage may have implications on differentiation potential, cell viability and more. Therefore, the proliferation enhancement method should be carefully validated within the context of the specific CM production process parameters, and regular monitoring of genomic integrity will likely have to be incorporated into the production process.

The burgeoning field of CM presents a unique set of challenges and opportunities related to cell proliferation and expansion. Basic biological questions surrounding cell division rate and methods to optimize it underscore the need for continuous research. The exploration of knowledge from pathological contexts may provide innovative solutions. As we embark on this transformative journey toward sustainable meat production, it becomes increasingly evident that addressing these multifaceted questions will be central to the success of CM.

## Data availability statement

The original contributions presented in the study are included in the article/supplementary material, further inquiries can be directed to the corresponding authors.

## Author contributions

MH: Conceptualization, Writing – original draft, Review & editing. AZ: Conceptualization, Writing – original draft, Review & editing. RR: Conceptualization, Writing – original draft, Review & editing. IN: Conceptualization, Writing – original draft, Review & editing.

## References

[1] Ben-Arye T Levenberg S. Tissue engineering for clean meat production. Front Sustain Food Syst. (2019) 3:46. doi: 10.3389/fsufs.2019.00046

[2] Post M Van der Weele C. Principles of tissue engineering for food In: Lanza R Langer R Vacanti J, editors. Principles of tissue engineering. Amsterdam, Netherlands: Elsevier (2014). 1647–62.

[3] Fraeye I Kratka M Vandenburgh H Thorrez L. Sensorial and nutritional aspects of cultured meat in comparison to traditional meat: much to be inferred. Front Nutr. (2020) 7:35. doi: 10.3389/fnut.2020.00035, PMID: 32266282 PMC7105824

[4] Olenic M Thorrez L. Cultured meat production: what we know, what we don’t know and what we should know. Ital J Anim Sci. (2023) 22:749–53. doi: 10.1080/1828051X.2023.2242702

[5] Kolodkin-Gal I Dash O Rak R. Probiotic cultivated meat: bacterial-based scaffolds and products to improve cultivated meat. Trends Biotechnol. (2023). doi: 10.1016/j.tibtech.2023.09.002, PMID: 37805297

[6] Post MJ Levenberg S Kaplan DL Genovese N Fu J Bryant CJ. Scientific, sustainability and regulatory challenges of cultured meat. Nat Food. (2020) 1:403–15. doi: 10.1038/s43016-020-0112-z

[7] Geay Y Bauchart D Hocquette JF Culioli J. Effect of nutritional factors on biochemical, structural and metabolic characteristics of muscles in ruminants, consequences on dietetic value and sensorial qualities of meat. Reprod Nutr Dev. (2001) 41:1–26. doi: 10.1051/rnd:2001108, PMID: 11368241

[8] Tziampazis E Sambanis A. Modeling of cell culture processes. Cytotechnology. (1994) 14:191–204. doi: 10.1007/BF00749616 7765590

[9] Hirsch HR Witten M. The waste-product theory of aging: simulation of metabolic waste production. Exp Gerontol. (1991) 26:549–67. doi: 10.1016/0531-5565(91)90073-u, PMID: 1800130

[10] Specht L. *An analysis of culture medium costs and production volumes for cultivated meat*. (2020).

[11] Messmer T Klevernic I Furquim C Ovchinnikova E Dogan A Cruz H. A serum-free media formulation for cultured meat production supports bovine satellite cell differentiation in the absence of serum starvation. Nat Food. (2022) 3:74–85. doi: 10.1038/s43016-021-00419-1, PMID: 37118488

[12] Stout AJ Mirliani AB Rittenberg ML Shub M White EC Yuen JSK. Simple and effective serum-free medium for sustained expansion of bovine satellite cells for cell cultured meat. Commun Biol. (2022) 5:466. doi: 10.1038/s42003-022-03423-8, PMID: 35654948 PMC9163123

[13] Nikkhah A Rohani A Zarei M Kulkarni A Batarseh FA Blackstone NT. Toward sustainable culture media: using artificial intelligence to optimize reduced-serum formulations for cultivated meat. Sci Total Environ. (2023) 894:164988. doi: 10.1016/j.scitotenv.2023.164988, PMID: 37343855

[14] Malumbres M Barbacid M. Cell cycle, CDKs and cancer: a changing paradigm. Nat Rev Cancer. (2009) 9:153–66. doi: 10.1038/nrc2602, PMID: 19238148

[15] Brandmaier A Hou S-Q Shen WH. Cell cycle control by PTEN. J Mol Biol. (2017) 429:2265–77. doi: 10.1016/j.jmb.2017.06.004, PMID: 28602818 PMC5659283

[16] Kippin TE Martens DJ van der Kooy D. p21 loss compromises the relative quiescence of forebrain stem cell proliferation leading to exhaustion of their proliferation capacity. Genes Dev. (2005) 19:756–67. doi: 10.1101/gad.1272305, PMID: 15769947 PMC1065728

[17] Plasilova M Schonmyer B Fernandez J Clavin N Soares M Mehrara BJ. Accelerating stem cell proliferation by down-regulation of cell cycle regulator p21. Plast Reconstr Surg. (2009) 123:149S–57S. doi: 10.1097/PRS.0b013e318191c82b, PMID: 19182674

[18] Jaluria P Betenbaugh M Konstantopoulos K Shiloach J. Enhancement of cell proliferation in various mammalian cell lines by gene insertion of a cyclin-dependent kinase homolog. BMC Biotechnol. (2007) 7:71. doi: 10.1186/1472-6750-7-71, PMID: 17945021 PMC2164945

[19] Vermeulen K Van Bockstaele DR Berneman ZN. The cell cycle: a review of regulation, deregulation and therapeutic targets in cancer. Cell Prolif. (2003) 36:131–49. doi: 10.1046/j.1365-2184.2003.00266.x, PMID: 12814430 PMC6496723

[20] Echave P Conlon IJ Lloyd AC. Cell size regulation in mammalian cells. Cell Cycle. (2007) 6:218–24. doi: 10.4161/cc.6.2.3744 17245129

[21] Ogura Y Sasakura Y. Emerging mechanisms regulating mitotic synchrony during animal embryogenesis. Develop Growth Differ. (2017) 59:565–79. doi: 10.1111/dgd.12391, PMID: 28833071

[22] Genovese NJ Desmet DN Schulze E. *Methods for extending the replicative capacity of somatic cells during an ex vivo cultivation process*. (2019).

[23] Genovese NJ Schulze EN Desmet DN. *Compositions and methods for increasing the efficiency of cell cultures used for food production*. (2022).

[24] Kumar SM Liu S Lu H Zhang H Zhang PJ Gimotty PA. Acquired cancer stem cell phenotypes through Oct4-mediated dedifferentiation. Oncogene. (2012) 31:4898–911. doi: 10.1038/onc.2011.656, PMID: 22286766 PMC3343184

[25] Slack JM. Stem cells in epithelial tissues. Science. (2000) 287:1431–3. doi: 10.1126/science.287.5457.1431 10688782

[26] Rak R Polonsky M Eizenberg-Magar I Mo Y Sakaguchi Y Mizrahi O. Dynamic changes in tRNA modifications and abundance during T cell activation. Proc Natl Acad Sci USA. (2021) 118. doi: 10.1073/pnas.2106556118, PMID: 34642250 PMC8594584

[27] Gingold H Tehler D Christoffersen NR Nielsen MM Asmar F Kooistra SM. A dual program for translation regulation in cellular proliferation and differentiation. Cell. (2014) 158:1281–92. doi: 10.1016/j.cell.2014.08.011, PMID: 25215487

[28] Itzkovitz S Blat IC Jacks T Clevers H van Oudenaarden A. Optimality in the development of intestinal crypts. Cell. (2012) 148:608–19. doi: 10.1016/j.cell.2011.12.025, PMID: 22304925 PMC3696183

[29] Maley CC Greaves M. Frontiers in Cancer research: Evolutionary foundations, revolutionary directions. Berlin: Springer (2016).

[30] Hanahan D. Hallmarks of cancer: new dimensions. Cancer Discov. (2022) 12:31–46. doi: 10.1158/2159-8290.CD-21-1059, PMID: 35022204

[31] Perona R. Cell signalling: growth factors and tyrosine kinase receptors. Clin Transl Oncol. (2006) 8:77–82. doi: 10.1007/s12094-006-0162-1 16632420

[32] Witsch E Sela M Yarden Y. Roles for growth factors in cancer progression. Physiology (Bethesda). (2010) 25:85–101. doi: 10.1152/physiol.00045.2009, PMID: 20430953 PMC3062054

[33] Pasitka L Cohen M Ehrlich A Gildor B Reuveni E Ayyash M. Spontaneous immortalization of chicken fibroblasts generates stable, high-yield cell lines for serum-free production of cultured meat. Nat Food. (2023) 4:35–50. doi: 10.1038/s43016-022-00658-w, PMID: 37118574

[34] Wang Y Chen S Yan Z Pei M. A prospect of cell immortalization combined with matrix microenvironmental optimization strategy for tissue engineering and regeneration. Cell Biosci. (2019) 9:7. doi: 10.1186/s13578-018-0264-9, PMID: 30627420 PMC6321683

[35] Hwang LN Yu Z Palmer DC Restifo NP. The in vivo expansion rate of properly stimulated transferred CD8+ T cells exceeds that of an aggressively growing mouse tumor. Cancer Res. (2006) 66:1132–8. doi: 10.1158/0008-5472.CAN-05-1679, PMID: 16424050 PMC1550975

[36] Shifrut E Carnevale J Tobin V Roth TL Woo JM Bui CT. Genome-wide CRISPR screens in primary human T cells reveal key regulators of immune function. Cell. (2018) 175:1958–1971.e15. doi: 10.1016/j.cell.2018.10.024, PMID: 30449619 PMC6689405

[37] Lewis DA Ly T. Cell cycle entry control in naïve and memory CD8+ T cells. Front Cell Dev Biol. (2021) 9:727441. doi: 10.3389/fcell.2021.727441, PMID: 34692683 PMC8526999

[38] Iconaru LI Ban D Bharatham K Ramanathan A Zhang W Shelat AA. Discovery of small molecules that inhibit the disordered protein, p27(Kip1). Sci Rep. (2015) 5:15686. doi: 10.1038/srep15686, PMID: 26507530 PMC4623604

[39] Mak LH Woscholski R. Targeting PTEN using small molecule inhibitors. Methods. (2015) 77-78:63–8. doi: 10.1016/j.ymeth.2015.02.007 25747336

[40] Shalem O Sanjana NE Hartenian E Shi X Scott DA Mikkelson T. Genome-scale CRISPR-Cas9 knockout screening in human cells. Science. (2014) 343:84–7. doi: 10.1126/science.1247005, PMID: 24336571 PMC4089965

[41] Marcu A Gaspar R Rutsaert P Seibt B Fletcher D Verbeke W. Analogies, metaphors, and wondering about the future: lay sense-making around synthetic meat. Public Underst Sci. (2015) 24:547–62. doi: 10.1177/0963662514521106, PMID: 24553438

[42] Soice E Johnston J. Immortalizing cells for human consumption. Int J Mol Sci. (2021) 22. doi: 10.3390/ijms222111660, PMID: 34769088 PMC8584139

[43] Juran CM Zvirblyte J Cheng-Campbell M Blaber EA Almeida EAC. Cdkn1a deletion or suppression by cyclic stretch enhance the osteogenic potential of bone marrow mesenchymal stem cell-derived cultures. Stem Cell Res. (2021) 56:102513. doi: 10.1016/j.scr.2021.102513 34517335

[44] Shen Y Zhang J Yu T Qi C. Generation of PTEN knockout bone marrow mesenchymal stem cell lines by CRISPR/Cas9-mediated genome editing. Cytotechnology. (2018) 70:783–91. doi: 10.1007/s10616-017-0183-3, PMID: 29387984 PMC5851970

[45] Kruse J-P Gu W. Modes of p53 regulation. Cell. (2009) 137:609–22. doi: 10.1016/j.cell.2009.04.050, PMID: 19450511 PMC3737742

[46] Armesilla-Diaz A Elvira G Silva A. p53 regulates the proliferation, differentiation and spontaneous transformation of mesenchymal stem cells. Exp Cell Res. (2009) 315:3598–610. doi: 10.1016/j.yexcr.2009.08.004, PMID: 19686735

[47] Li X Bai J Ji X Li R Xuan Y Wang Y. Comprehensive characterization of four different populations of human mesenchymal stem cells as regards their immune properties, proliferation and differentiation. Int J Mol Med. (2014) 34:695–704. doi: 10.3892/ijmm.2014.1821, PMID: 24970492 PMC4121354

[48] Lyons AB Parish CR. Determination of lymphocyte division by flow cytometry. J Immunol Methods. (1994) 171:131–7. doi: 10.1016/0022-1759(94)90236-4 8176234

[49] Yoon H Kim TS Braciale TJ. The cell cycle time of CD8+ T cells responding in vivo is controlled by the type of antigenic stimulus. PLoS One. (2010) 5:e15423. doi: 10.1371/journal.pone.0015423, PMID: 21079741 PMC2975678

